# 肺癌脑转移的诊治进展

**DOI:** 10.3779/j.issn.1009-3419.2013.07.10

**Published:** 2013-07-20

**Authors:** 懿 刘, 军 陈

**Affiliations:** 300052 天津，天津医科大学总医院肺部肿瘤外科 Department of Lung Cancer Surgery, Tianjin Medical University General Hospital, Tianjin 300052, China

**Keywords:** 肺肿瘤, 脑转移, 诊断, 治疗, Lung neoplasms, Brain metastasis, Diagnosis, Treatment

## Abstract

肺癌脑转移的发生率为23%-65%，是脑转移性肿瘤中最常见的类型，且预后较差。目前肺癌脑转移的诊治和分子机理已成为众多研究的热点之一。本文就肺癌脑转移的临床特征、诊断和治疗方面的进展以及最新脑转移的机制学研究做了系统的综述。

## Abstract (en)

Lung cancer with brain metastasis was 23% to 65%, and is the most common type in brain metastasis tumors with the poor prognosis. At present, diagnosis and treatment of brain metastases from lung carcinoma and its molecular mechanism have become one hot spot of amount researches. Here, we made a systematic review of the progress of the clinical features, diagnosis and treatment of brain metastases from lung and its molecular mechanism.

肺癌最常见的远处转移部位之一是脑部，肺癌脑转移的发生率为23%-65%，是脑转移性肿瘤中最常见的类型^[1]^。随着肺癌发病率的逐年上升，各种诊疗技术的提高，肺癌脑转移的检出率也随之增加。肺癌脑转移预后较差，其中位生存期为3.1个-12个月^[2]^。目前关于肺癌脑转移的诊断和治疗成为众多研究的热点之一，不断有新方法新手段涌现出，且效果各有不同，现将其进展综述如下。

## 1 肺癌脑转移的临床症状

肺癌脑转移的临床症状与转移瘤所在部位处神经的功能以及肿瘤的大小密切相关。临床上常见的症状有逐步加剧的头痛，可伴有恶心呕吐、肢体运动及感觉障碍、失语、头晕、记忆力减退等等，也可以无任何症状^[3]^。除了以上这些常见的转移瘤症状外，此外，亦有一些不常见的个例报道，如Hui等^[4]^报道了1例19岁女孩首先发现颅脑孤立病灶，手术切除后诊断为转移性小细胞肺癌（small cell lung cancer, SCLC），并且通过经气管镜穿刺活检，肺部也诊断为小细胞肺癌。这是迄今文献报道年龄最小的首先以脑转移诊断的肺癌患者。松果体区域不是常见脑转移部位，且此处的转移一般亦没有症状，但Samanci等^[5]^报道了1例53岁肺癌患者出现剧烈头痛，复视，进一步检查及手术证实了为肺癌松果体处转移。Ray等^[6]^报道了1例诊为Lambert-Eaton肌无力综合症的患者，其头部有孤立肿瘤，手术切除后病理提示转移性小细胞肺癌，但进一步的检查却没有发现明确的肺部原发病灶。

## 2 肺癌脑转移的诊断

影像学检查中，头颅CT可见低密度病灶，等密度及高密度病灶比较少见，增强现象明显。头颅核磁共振成像平扫可见典型长T1、长T2异常信号，病灶周围不规则水肿，一般来说，病灶体积越大，水肿越明显，增强扫描后可见病灶周围环形强化。对于考虑头部存在转移的肺癌患者，两倍剂量强化的头颅核磁共振成像是推荐的必要检查，因其可以检测到CT检查所不能发现的微小转移灶^[7]^。近年来，PET/CT在肺癌分期中的应用越来越广泛，有学者对其在颅脑转移中的检测做了一项回顾性的研究，其包括56项研究组和8, 699例肺癌患者，分析发现PET/CT对脑转移诊断的敏感性较低，为26.5%^[8]^。在另一组104例临床无神经症状的肺癌患者的研究中，同时应用PET/CT和头部MRI检测脑转移，PET/CT对于脑转移诊断的敏感性仅为27.3%，特异性为97.6%。而且57%的用PET/CT检测到的脑转移，其肿瘤直径均大于在MRI测量直径，故认为MRI检测脑转移比PET/CT更准确^[9]^。此外，在一项关于选择性头颅PET/CT在小细胞肺癌中的应用研究^[10]^表明，选择性头颅PET/CT对于小细胞肺癌脑转移的诊断是有可利用临床价值的。目前，虽然有多种肺癌辅助诊断的肿瘤标志物不断涌现出，但真正对转移检测有价值的标志物还没有确定。Lee等^[11]^报道在227例晚期肺癌患者血清中联合检测CEA、CYFRA 21-1、CA125、CA19-9和SCC的表达发现，只有CEA的表达在有无脑转移的肺癌患者的血清中具有显著性差异（*P*=0.000, 1），从而提示CEA在肺癌脑转移过程中发挥重要作用，对于晚期肺癌应该监测血清中CEA的变化。此外，还有研究^[12]^提示E-cadherin可以作为非小细胞肺癌（non-small cell lung cancer, NSCLC）脑转移的标志物。近年来亦出现MRI-PET在肺癌脑转移诊断中的作用研究，如一项143例NSCLC患者研究中，随机应用MRI-PET对比PET-CT联合颅脑MRI检测脑转移，发现MRI-PET在脑转移中并不具有更大的优势^[13]^。除了明显肿块外，脑转移的影像表现亦有为多发的小的钙化结节^[14, 15]^。

## 3 肺癌脑转移的基础研究

肺癌细胞侵入临近血管和淋巴管，很容易经过体循环到达远处器官形成转移。因脑的血供量约占全部体血循环的1/5，故发生转移癌细胞的机会较多。在肺癌脑转移的基础研究方面，近年来的研究报道较多。有研究^[16]^表明在脑转移的肺癌患者中，CXCR4蛋白存在过表达，提示高表达CXCR4与肺癌脑转移相关。在以特异性脑转移肺癌细胞株PC14/B为研究对象的研究中发现，抑制该高表达的S100B蛋白后，细胞增殖和迁移功能明显降低，从而提示S100B蛋白的表达与脑转移的存在密切相关^[17]^。microRNAs调控目标RNA的表达，在肺癌脑转移中也有相应研究，有研究^[18, 19]^认为microRNA-328、microRNA-378在肺癌脑转移中起着重要作用。亦有研究^[20]^表明在SCLC患者血清中，高水平表达胎盘生长因子（placenta growth factor, PLGF）的患者更易于发生脑转移; 应用体外血脑屏障模型，进一步证实了PLGF可以使SCLC细胞透过血脑屏障，当抑制了PLGF的表达后，在模型中会抑制体外SCLC细胞发生脑转移的几率。巢蛋白（nestin）是一种中间丝类型的蛋白，对神经元分化有作用，是神经干细胞的特征性标志物。SKARDA等^[21]^用免疫组化检测114例NSCLC标本，发现在发生脑转移的肺鳞癌中表达明显高于肺腺癌。

## 4 全脑照射（whole-brain radiotherapy, WBRT）

WBRT被广泛应用于治疗肺癌脑转移，总体来说，WBRT可以快速缓解神经症状，提高患者生存期。WBRT对于多发脑转移作用较好。对于手术切除后或者立体定向放疗后的患者，WBRT也可以用来作为其后的辅助治疗。关于WBRT同时联合全身化疗还是序贯性治疗更使患者受益的问题，有报道^[22]^将60例确诊为肺癌脑转移患者分为两组，一组为同时组即同时联合WBRT和全身化疗，另一组为序贯组即这两种治疗序贯性进行，两组总体反应率22%，脑部反应率35.6%，中位PFS 3个月，总体患者1年、2年生存率分别为55%和24.4%;中位生存期16个月。在两组中，总体反应率、脑部反应率、中位生存期和1年生存期没有明显差异，但2年生存率同时组为37.2%，序贯组为18.9%（*P*=0.011），并且序贯组的粒细胞减少发生几率高于同时组（*P*=0.029）。在另一项针对肺癌的原发肿瘤治疗研究^[23]^中，30例患者先经过WBRT，然后经过两周期紫杉醇加顺铂化疗，如果病情没有进展的话，对于肺部原发肿瘤进行放化疗同时治疗，方案为紫杉醇加卡铂，放疗剂量为60 Gy，如果病情进展的话将会排除出组进行4个周期的化疗。中位PFS和OS分别为8.43个月和31.8个月，1年和2年OS率分别为71.1%和60.2%。对于分期在N0-N1的3年生存率明显高于N2-N3患者。以前的临床研究认为对于通过放化疗CR的SCLC应该给予预防性全脑照射，但对于放化疗反应不佳的SCLC是否可以从预防性全脑照射中受益，在近期研究中已经获得证实，预防性全脑照射可以明显降低脑转移的发生^[24, 25]^。

## 5 立体定向放射治疗（stereotactic radiosurgery, SRS）

立体定向放射治疗具有定位精确、剂量集中和安全快速等特点，并且具有非侵袭性、损伤相对较小和恢复快等优点，可以快速控制局部肿瘤进展，缓解神经系统症状。有研究^[26]^对于可手术切除的NSCLC脑转移的64例患者应用伽玛刀治疗，平均生存期为13.5个月，平均OS为31.5个月，总结认为对于存在3个及以下脑部转移灶的NSCLC，伽玛刀是有效的。Abacioglu等^[27]^总结了100例经过伽玛刀治疗的NSCLC患者，其中78例患者在伽玛刀治疗前后经过WBRT，有26例经过手术切除脑部转移灶，从伽玛刀治疗开始中位生存期9个月，从诊断脑转移开始中位生存期为14个月。局部肿瘤控制率达到95%。伽玛刀治疗时间的选择对于患者的受益情况也是有区别的，Rahn等^[28]^将NSCLC脑转移患者分为两组，一组上午进行治疗，另一组选择下午治疗，结果发现前者局部控制率为97%，而后者为67%（*P*=0.014）。Harris等^[29]^在一项确诊脑转移的51例经过WBRT失败的SCLC患者中，应伽玛刀作为姑息治疗，可以使患者从中受益。Nakazaki等^[30]^总结了44例病例，亦得出了相同的结论。对于没有临床症状的，初次诊断为脑转移的肺癌患者中，有研究比较了应用伽玛刀和其它方法治疗脑部转移灶，提示单独应用伽玛刀治疗和其它标准方案治疗比较，患者受益率很接近^[31]^。在一项大分割立体定向放射治疗初次诊断为脑转移的NSCLC中，通过对171例患者治疗观察，大分割立体定向放射治疗的整体生存率和其它治疗相当^[32]^。

## 6 手术治疗

对于单发脑转移，手术是很重要的一项治疗方式。手术可以快速缓解肿瘤压迫症状并且取得确定的病理诊断，可以做到有效的局部控制。存在肿瘤压迫造成致命性的病变，手术切除是最佳的选择。一组17例肺癌伴单发脑转移病例中，同时行肺部手术及神经外科手术切除病灶，中位生存期52个月，5年生存率27%^[33]^。在另一组数据中，41例单发脑转移的肺癌患者经过神经外科手术治疗的中位生存期15.4个月，而经过WBRT的患者中位生存期仅为11.5个月（*P*=0.002）^[34]^。在另一个单中心的研究中，31例伴有脑转移的NSCLC中，26例有1处转移灶，另外5例有2处转移灶。10例患者行神经外科手术治疗，另外20例患者行SRS，还有1例患者同时用以上两种方法治疗。27例患者肺部肿瘤完整切除，4例患者姑息性切除。1年、2年和5年生存率分别为83%、47%和21%。中位生存期为22个月，认为对于有选择的患者，用联合的方法治疗合并脑转移的肺癌患者可以取得满意效果^[35]^。

## 7 化疗

传统观点认为化疗药物难以通过血脑屏障，对脑转移的治疗效果有限。但近期研究表明肺癌发生脑转移血脑屏障已有破坏，存在一定的通透性。许多化疗药物可以透过血脑屏障进入脑内，发挥抗肿瘤作用。在一项培美曲塞联合顺铂同时行WBRT治疗肺腺癌脑转移的临床研究中，入组42例患者，经过6个周期培美曲塞加顺铂化疗，同期行WBRT结果证实患者可以从中受益^[36]^。关于用培美曲塞作NSCLC脑转移的维持治疗，也有2例报道，这2例患者脑部肿瘤复发，在经过立体定向放射治疗后，以培美曲塞作维持治疗，经过2年随访，没有肿瘤复发^[37]^。Rades等^[38]^报道1例联合应用放射标记的西妥西单抗和WBRT治疗NSCLC多发脑转移患者，颅内最大转移灶从治疗前的40 mm×33 mm×27 mm减小为31 mm×22 mm×21 mm。

## 8 分子靶向治疗

分子靶向治疗已成为肺癌治疗的重要手段，分子靶向药物为肺癌脑转移提供了新的方法。Park等^[39]^在存在*EGFR*受体突变的一组28例发生脑转移的NSCLC中，应用厄洛替尼或者吉非替尼治疗，23例患者为部分缓解，3例为疾病稳定，疾病控制率达到93%，PFS和OS分别为6.6个月和15.9个月。在一项厄洛替尼联合WBRT的临床研究^[40]^中，40例诊为脑转移的NSCLC患者同期以厄洛替尼和WBRT治疗，中位随访时间28.5个月，中位生存期11.8个月，其中野生型*EGFR*中位生存期为9.3个月，*EGFR*突变的患者中位生存期为19.1个月。Porta等^[41]^在69例诊断为脑转移的NSCLC患者中检*EGFR*受体基因突变情况，其中17例患者存在外显子19或者21突变，应用厄洛替尼治疗，存在突变患者反应率82.4%，存在*EGFR*突变的患者中位生存期12.9个月，未突变的患者中位生存期3.1个月（*P* < 0.001）。一组吉非替尼联合放疗作为治疗手段的研究中，38例肺癌脑转移患者口服吉非替尼联合脑部放疗作为观察组，123例患者单用放疗作为对照组，比较两组患者，反应率分别为31.6%和15.4%（*P*=0.027），疾病控制率分别为78.9%和60.2%（*P*=0.034）。观察组治疗后的PS评分明显高于对照组（*P*=0.044），认为吉非替尼联合放疗治疗NSCLC脑转移可以提高疗效和患者生活质量^[42]^。曾有观点认为肺癌脑转移患者使用TKI治疗会增加中枢神经出血的几率，Sandler等^[43]^经过复习文献，认为使用TKI药物不会增加中枢神经系统出血几率，是一种安全的治疗肺癌脑转移的方法。目前存在*EGFR*受体突变的脑转移的NSCLC患者中，应用TKI药物的反应率为70%-80%，已经成为治疗脑转移的重要的手段之一^[44]^。

## 9 预后

有报道^[45]^分析了290经历各种治疗的肺癌脑转移患者，中位生存期为14个月，1年、2年和3年生存率分别为56%、28.3%和12%，指出KPS≥70分、病理类型为腺癌和长期应用TKI药物是影响预后的因素。有学者^[46]^报道了186例首先诊断脑部肿瘤后来确诊为肺癌转移的患者，中位生存时间为15个月。肺癌脑脊膜转移恶性程度很高，病情进展快，Morris等^[47]^总结了125例此类患者，中位生存期只有3个月，WBRT的患者没有效果，经过鞘内化疗的患者，中位生存期达到18个月，存在*EGFR*突变并应用TKI类药物患者中位生存期达到14个月。

## 10 展望

原发性肿瘤的致死率约占10%，而肿瘤转移致死率约90%。大多数的脑转移来自于肺癌，对于其发生机制及治疗方式的研究仍在继续，不断有新的理论和方法问世并应用于临床。比如最新研究应用包括神经干细胞表达自杀基因，导入血脑屏障，杀灭颅脑转移病灶，在动物实验中取得初步成果^[48]^，还需要进一步研究。
